# An animal model of limitation of gut colonization by carbapenemase-producing *Klebsiella pneumoniae* using rifaximin

**DOI:** 10.1038/s41598-022-07827-8

**Published:** 2022-03-08

**Authors:** Eleni Xenofontos, Georgios Renieris, Maria Kalogridi, Dionyssia-Eirini Droggiti, Kalliopi Synodinou, Georgia Damoraki, Panagiotis Koufargyris, Labros Sabracos, Evangelos J. Giamarellos-Bourboulis

**Affiliations:** 1 4th Department of Internal Medicine, National and Kapodistrian University of Athens, Medical School, Athens, Greece; 2 4th Department of Internal Medicine, ATTIKON University Hospital, 1 Rimini Street, 124 62 Athens, Greece

**Keywords:** Drug discovery, Microbiology, Diseases, Infectious diseases

## Abstract

Current knowledge suggests that infection by carbapenem-resistant enterobacteria is preceded by gut colonization. It is hypothesized that colonization is eradicated by non-absorbable antibiotics like rifaximin. We investigated the effect of rifaximin against carbapenem-resistant *Klebsiella pneumoniae* (CRKP) in vitro and in a mouse model. We studied the in vitro efficacy of rifaximin against 257 CRKP clinical isolates, 188 KPC producers and 69 OXA-48 producers, by minimum inhibitory concentration and time-kill assays. We then developed a model of gut colonization by feeding 30 C57Bl6 mice with 10^8^ cfu of one KPC-KP isolate for 7 days; mice were pre-treated orally with saline, omeprazole or ampicillin. Then, another 60 mice with established KPC-2 gut colonization received orally for 7 consecutive days rifaximin 180 mg/kg dissolved in ethanol and 4% bile or vehicle. On days 0, 3 and 7 stool samples were collected; mice were sacrificed for determination of tissue outgrowth. At a concentration of 1000 μg/ml rifaximin inhibited 84.8% of CRKP isolates. Α 3 × log_10_ decrease of the starting inoculum was achieved by 100, 250 and 500 μg/ml of rifaximin after 24 h against 25, 55 and 55% of isolates. Pre-treatment with ampicillin was necessary for gut colonization by KPC-KP. Treatment with rifaximin succeeded in reducing KPC-KP load in stool and in the intestine. Rifaximin inhibits at clinically meaningful gut concentrations the majority of CRKP isolates and is efficient against gut colonization by KPC-KP.

## Introduction

Enterobacteriaceae are one of the leading causes of healthcare-associated infections in Greece^1^. This holds true for infection by carbapenem-resistant *Klebsiella (K.) pneumoniae* (CRKP). Their emergence is associated with mortality up to 50%^2^. Recent epidemiology studies report prevalence of these species ranging in Europe between less than 1% in the Scandinavian countries, to 30% in Italy, and more than 50% in Greece^3^ and China^4^ but also ranging between 2.4 and 50.8% in the United States^5^. These isolates infect debilitated hosts like patients hospitalized in the Intensive Care Units (ICUs) and patients with hematologic malignancies. It appears that intestinal colonization is the major reservoir for these resistant isolates among critically ill patients^6,7^ and patients with hematologic malignancies^8^. Thus, the eradication of gut colonization by carbapenem resistant enterobacteriaceae (CRE) is an attractive strategy for infection prevention.

Rifaximin is a non-absorbable, rifamycin derivate with a broad antimicrobial spectrum. Oral treatment with rifaximin has been demonstrated to lead to high intra-intestinal concentrations even exceeding 8000 μg/g; these are attained after three days of oral intake of 400 mg rifaximin per day^9,10^. Due to its non-absorbable nature, rifaximin has been used in the management of travellers’ diarrhea and hepatic encephalopathy^11,12^ and more recently for treating irritable bowel syndrome^10,13,14^. Our group has shown considerable in vitro efficacy of rifaximin against susceptible isolates of *Klebsiella* spp.^15^ generating the hypothesis if rifaximin may be a candidate drug for the eradication of CRE.

The present study has two stages. At the first stage, we investigated the in vitro activity of rifaximin against isolates of CRE species of KP. In the second stage, we investigated the efficacy of rifaximin in the eradication of gut colonization by CRE producing KPC in an animal model of gut colonization.

## Results

### In vitro susceptibility testing

A total of 257 CRE clinical isolates from specimens of blood (n = 128), urine (n = 76), tracheobronchial secretions (n = 25), rectal swab (n = 10) and pus (n = 18) were studied; 188 isolates harbored *bla*_KPC_ and 69 isolates harbored *bla*_OXA-48_-like.

Susceptibility testing results for all tested antimicrobials are shown in Table 1. In the absence of EUCAST MIC breakpoint for rifaximin against *K. pneumoniae*, we chose the clinically meaningful concentration of 1000 μg/ml, which is 1/8 of the fecal concentration that is achieved after oral administration^9,10^. At this concentration rifaximin inhibited in vitro 84.8% of isolates. At this concentration, rifaximin inhibited 84.0% and 86.9% of KPC and OXA-48-like producing isolates respectively (Table 1). Among the tested isolates with MIC > 1000 μg/ml, 30 (71.4%) harbored *bla*_KPC_ and 12 (28.6%) harbored *bla*_OXA-48_. 64.2% of these isolates were isolated from blood specimens, 14.3% from urine, 14.3% from TBS and the rest 7.1% from pus or rectal swab.

**Table 1:** In vitro activity of rifaximin and comparator antimicrobial agents against 257 *K. pneumoniae* clinical isolates according to their resistance phenotype.

Phenotype	Antimicrobial agent	MIC (μg/ml)			% of inhibited isolates
		Range	MIC_50_	MIC_90_	
KPC (n = 188)	Amikacin (8)	1 to > 128	> 128	> 128	11.7
	Meropenem (8)	8 to > 32	32	> 32	0
	Tigecycline (2)	0.06 to > 32	2	> 32	74.4
	Colistin (≤ 2)	0.015 to > 32	16	> 32	45.7
	Rifaximin	8 to > 2000	125	2000	84.0
OXA-48 (n = 69)	Amikacin (8)	8 to > 128	> 128	> 128	10.1
	Meropenem (8)	8 to > 32	32	> 32	0
	Tigecycline (2)	0.015 to > 32	2	> 32	79.7
	Colistin (≤ 2)	0.03 to > 32	16	> 32	34.8
	Rifaximin	32 to > 2000	125	2000	86.9

*EUCAST* European Committee on Antimicrobial Susceptibility Testing, *MIC* minimum inhibitory concentration, *MIC50/90* MIC required to inhibit 50% and 90% of the isolates, respectively.

The EUCAST breakpoint is provided in parentheses, where available. For Tigecycline the epidemiological cut-off value (ECOFF) is provided.

The breakpoint for rifaximin was set to a clinically meaningful concentration of 1000 μg/ml.

**Figure 1:**
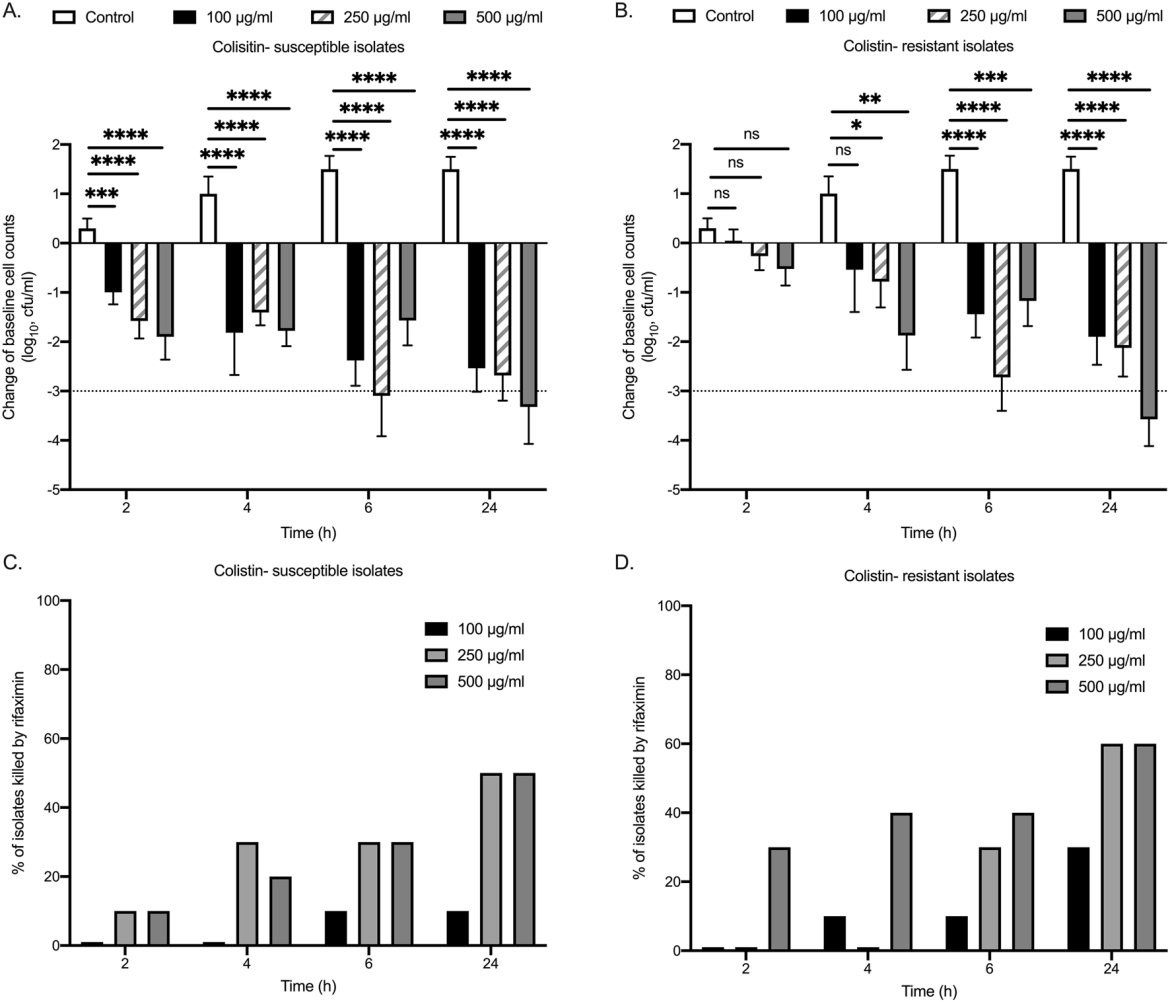
Time–kill effect of rifaximin against *Klebsiella pneumoniae* producing carbapenemase (KPC-KP) isolates. 20 KPC-KP isolates with minimal inhibitory concentration (MIC) from 8 to 128 μg/ml were exposed over time with/ without rifaximin in a concentration of 100, 250 and 500 μg/ml for 24 h. Cumulative change in the growth of colistin-susceptible (**A**) and colistin-resistant (**B**) isolates after exposure to different concentrations of rifaximin. Percentage of colistin-susceptible (**C**) and colistin resistant (**D**) isolates eradicated by rifaximin in different concentrations. Comparison by the Mann–Whitney-U test; ns non-significant, *p < 0.05, **p < 0.01, ***p < 0.001, ****p < 0.0001.

**Figure 2:**
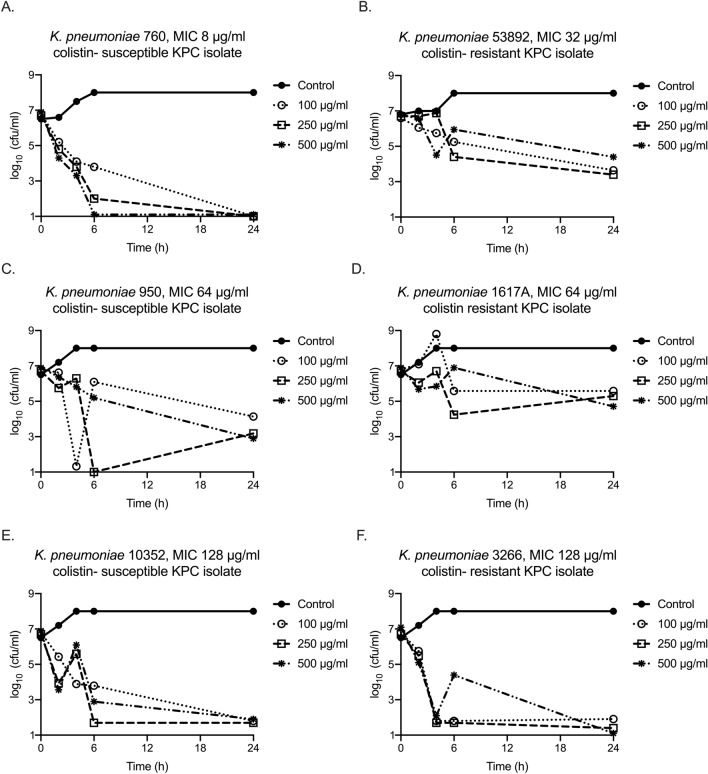
Time–kill effect of rifaximin against *Klebsiella pneumoniae* producing carbapenemase (KPC-KP) isolates according to minimal inhibitory concentration and susceptibility to colistin. Effect of rifaximin on (**A**) colistin-susceptible KPC-KP isolate 760 with MIC of rifaximin 8 μg/ml; (**B**) colistin-resistant KPC-KP isolate 53892 with MIC of rifaximin 32 μg/ml; (**C**) colistin-susceptible KPC-KP isolate 950 with MIC of rifaximin 64 μg/ml; (**D**) colistin-resistant KPC-KP isolate 1617A with MIC of rifaximin 64 μg/ml; (**E**) colistin-susceptible KPC-KP isolate 10352 with MIC of rifaximin 128 μg/ml; and (**F**) colistin-resistant KPC-KP isolate 3266 with MIC of rifaximin 128 μg/ml.

To validate the use of rifaximin we conducted time-kill assays of rifaximin against 10 CRKP isolates susceptible to colistin and 10 CRKP isolates resistant to colistin. MICs of rifaximin for the 20 CRKP isolates used for the time-kill assays ranged between 8 and 2000 μg/ml. The effect of rifaximin was concentration- and time-dependent for both colistin-susceptible and colistin-resistant isolates (Fig. 1A,B). A bactericidal effect was observed by 100, 250 and 500 μg/ml of rifaximin after six hours of exposure against 15, 30 and 35% of isolates and after 24 h of exposure against 25, 55 and 55% of isolates (Fig. 1C,D). Figure 2A–F show the characteristic time-kill effect on 3 colistin-susceptible and 3 colistin-resistant isolates with different MICs.

### Animal study

**Figure 3:**
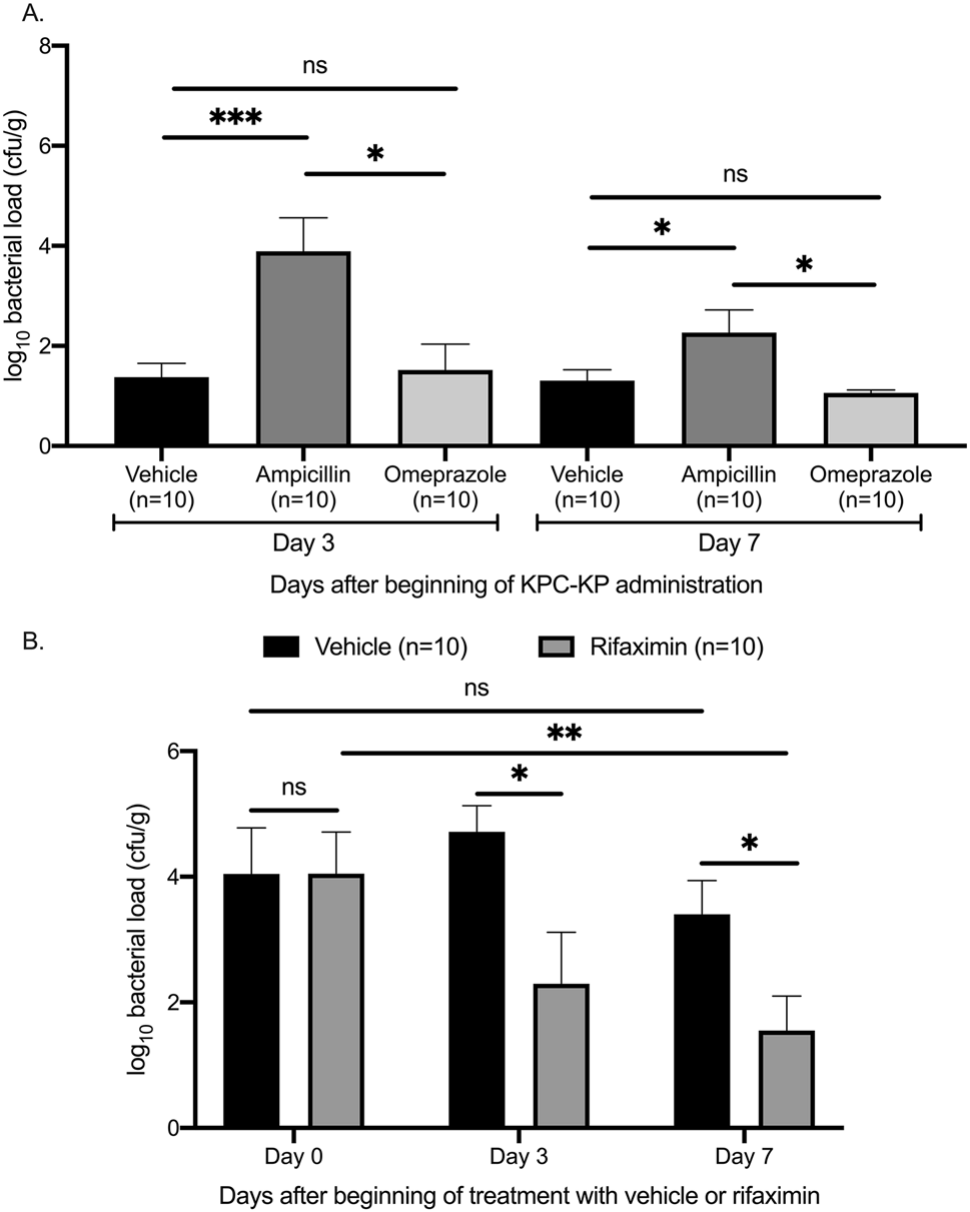
Effect of rifaximin on outgrowth of *Klebsiella pneumoniae* producing carbapenemase (KPC-KP) in stool. (**A**) Establishment of an efficient experimental model for gut colonization by KPC-KP. C57Bl6 mice were pre-treated orally with saline, omeprazole or ampicillin for 5 days and then fed with 10^8^ cfu KPC-KP for 7 days. KPC-KP growth was measured in stool 3 and 7 days after beginning of KPC-KP administration. (**B**) C57Bl6 mice with established KPC-KP gut colonization were treated orally with rifaximin or vehicle. KPC-KP growth was measured in stool 0, 3 and 7 days after beginning of rifaximin treatment. Comparison by the Mann–Whitney-U test; ns non-significant, *p < 0.05, **p < 0.01, ***p < 0.001.

The promising results of the in vitro studies and of the time-kill assays led us to investigate the value of rifaximin for the elimination of gut colonization by KPC-producing *K. pneumoniae* (KPC-KP) in vivo. Originally, we developed a mouse model of gut colonization by one species producing KPC. We chose to study one isolate of *K. pneumoniae* producing KPC-2 due to the predominance of this mechanism of resistance in the Mediterranean countries^16^. The attempt to colonize the gut of C57Bl6 mice simply by oral administration of KPC-KP was unsuccessful (Fig. 3A). Therefore, we tested the effect of pretreatment with the proton-pump inhibitor omeprazole or with ampicillin, which are known to influence gut microbiota and facilitate intestinal colonization by pathogenic bacteria^17,18^. Pretreatment with ampicillin but not with omeprazole led to sufficient growth of KPC-KP in stool 3 and 7 days after start of KPC-KP gavage (Fig. 3A). This model was chosen for further experimentation.

**Figure 4:**
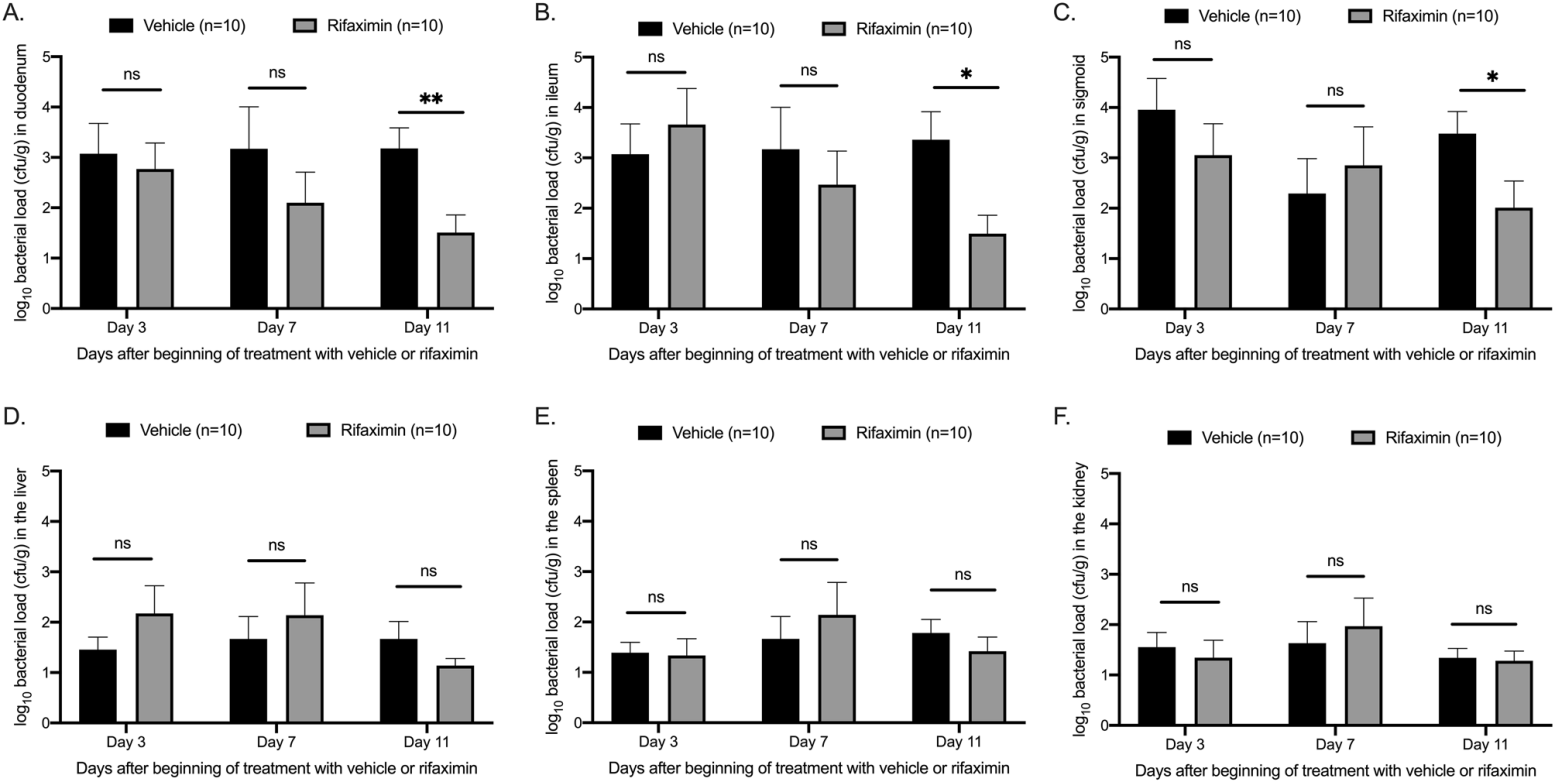
Effect of rifaximin on tissue outgrowth of *Klebsiella pneumoniae* producing carbapenemase (KPC-KP). C57Bl6 mice with established KPC-KP gut colonization were treated orally with rifaximin or vehicle. KPC-KP growth was determined 3, 7 and 11 after beginning of treatment in the duodenum (**A**), ileum (**B**), sigmoid (**C**); liver (**D**), spleen (**E**) and kidney (**F**). Comparison by Mann–Whitney-U test; ns non-significant, *p < 0.05, **p < 0.01.

Following establishment of gut colonization, oral treatment with rifaximin started for seven days. KPC-KP loads were significantly reduced in stool after 3 and 7 days compared to vehicle-treated mice (Fig. 3B). Moreover, after 7 days of treatment with rifaximin bacterial loads in samples of the duodenum, ileum and colon were significantly reduced compared to samples from mice treated with vehicle (Fig. 4A–C). The KPC-KP growth in the intrabdominal organs of mice was minimal (Fig. 4D–F). Mice did not show any signs of systemic disease like agitation or denial for food intake. Nil mouse died before the planned time of sacrifice.

## Discussion

The results of the present study clearly indicate a fair in vitro activity of rifaximin against CRKP isolates involving major resistance phenotypes i.e. KPC-KP and OXA-48-like-KP. Rifaximin was bactericidal against both colistin-susceptible and colistin-resistant isolates.

CRE have emerged as important multidrug-resistant nosocomial pathogens worldwide. In some countries like Italy, Greece and some parts of the United States, CRKP represents a significant proportion (ca. 40%) of *K. pneumoniae* isolates^1,19^. Moreover, CRKP has become a major clinical concern owing to their rapidly increasing resistance to nearly all currently available antibiotics. Gut is considered the main reservoir for these species. The importance infectious disease practitioners give to gut colonization is coming from two major situations: (a) the need to know gut CRE carriage sampling at regular time intervals from the time of admission in the ICU to guide empiric therapy in case of emergence of infection^20^; and (b) several case-reports suggesting the attempt for oral eradication with antibiotics like gentamicin and cefotaxime^21^. The achieved limitation of CRE outgrowth in the gut with this approach is ranging between 71 and 96%^22,23^.

Eradication of CRE colonizing the gut is a clinical necessity. CRE colonization is associated with 10.8 odds ratio for new infection by CRE and with 2.8 odds ratio for death compared to non-colonized patients^24^. These risks are unique for patients colonized by CRE and not for patients colonized by *Enterobacteriaceae* resistant to cephalosporins or other β-lactams whatever enlightens the need of development of strategies of selective oral decontamination (SOD). One published meta-analysis included 11 studies of SOD against multidrug-resistant (MDR) *Enterobacteriaceae* and did not show any benefit^25^ underlying how difficult eradication is. Two randomized clinical trials (RCTs) including limited number of patients colonized by MDR *Enterobacteriaceae* not-resistant to carbapenems showed that the use of either oral colistin/neomycin or short antibiotic treatment followed by fecal transplantation achieved temporary suppression of intestinal carriage; re-growth was observed following end of treatment^26,27^. In a prospective study, 77 patients colonized by CRE received oral gentamicin or an oral combination of neomycin and streptomycin for two weeks; decolonization was achieved in 44 patients. The risk of de-colonized patients to die the next 180 days was significantly lower than patients remaining colonized^28^ and this outscores the necessity to achieve gut eradication by CRE no matter how difficult this may be. Oral rifaximin had been used to eradicate gut colonization by MDR *Enterobacteriaceae*. In a retrospective analysis, oral rifaximin for two to three weeks was more effective (60% eradication rate among 15 treated patients) compared to low-dose oral colistin (39% eradication rate among 18 treated patients) and to high-dose oral colistin (25% eradication rate among 12 treated patients)^29^.

The real unmet need is the lack of any existing, so far, program of development of a strategy of CRE elimination from the gut. This program should involve animal studies and large-scale RCTs. Our study is the first animal model aiming to explore the possibility that an oral non-absorbable antibiotic restrains CRE gut colonization. The model is using previous treatment with one β-lactam which is altering the composition of gut microbiota^30^ and favors gut colonization by KPC. Oral treatment with rifaximin in this model is efficient in decreasing KPC load in the intestine and in stool of mice with already established gut colonization by KPC-2. Gut colonization was reduced but not fully eliminated. This introduces the concern how promising in the clinical setting rifaximin may be. The data presented herein favour the development of rifaximin for the eradication of CRE gut decolonization when analysed in the context of the existing clinical evidence. Non-achieving full eradication of CRE from the gut may be associated to the following (i) the duration of treatment should last for more than seven days; (ii) strategies of SOD are not similarly effective to all patients and CRE eradication has been shown difficult to be achieved; (iii) animal results allow to have precise quantitative cultures in both the levels of the stool and of the tissue. This is far different than clinical studies evaluating gut colonization through rectal swab cultures which have a threshold of positivity greater than the precise quantitative techniques^31^. Furthermore, clinical studies do not provide information of CRE colonizing the gut at different levels, as we did in our animal model.

The exact extrapolation of the findings for the human situation may be uncertain. However, the clinical aim is to decrease gut colonization to limit the risk of infections. Rifaximin possesses several advantages over comparators making it an attractive choice for use in clinical practice. At first, rifaximin has an excellent safety profile and tolerability with limited systemic absorption^10,32^. At second, it does not affect the composition of normal flora. In a recent randomized study, 36 patients with decompensated cirrhosis were administered rifaximin for 4 weeks. Rifaximin intake marginally reduced the abundance of the gut flora as this was expressed by the Shannon diversity index compared to the 18 placebo-treated patients; no effect on markers of systemic inflammation was found^33^. One limitation could be poor solubility in aqueous solvents^34^. These drug characteristics have led others to consider that rifaximin should become part of the antimicrobials that are used for selective decontamination of the digestive tract^35^.

In conclusion, this study demonstrates rifaximin as a potential adjunctive treatment against gut colonization by CRE. Further experimental studies are needed for the validation of the use of rifaximin against CRE and other resistant strains associated with intestinal colonization before translational efforts centered on the use of rifaximin against resistant pathogens can be initiated.

## Materials and methods

### In vitro study

Stored CRKP in skim-milk at − 80 °C were used for this study. All were isolated from biological samples of patients with documented hospital-acquired infections aggravated by systemic inflammatory response syndrome and hospitalized between September 2014 and December 2017 in four departments of Internal Medicine and two Intensive Care Units participating in the Hellenic Sepsis Study Group (http://www.sepsis.gr). The study protocol was approved by the Ethics Committees of the participating hospitals. Patients were enrolled after written informed consent provided by themselves or by first degree relatives in case of patients unable to consent. Isolates were shipped to the central lab at the Laboratory of Immunology of Infectious Diseases of the 4th Department of Internal Medicine at ATTIKON University Hospital for further testing. All methods were performed in accordance with the relevant guidelines and regulations. Resistance to carbapenems was determined by measurement of MICs to meropenem with the microdilution technique. Resistance genotype of KPC or OXA-48-like production was determined by polymerase chain reaction^36^.

MICs of amikacin, meropenem, tigecycline, colistin and rifaximin were determined by the broth microdilution technique in a final volume of 0.1 ml of MHB using one log-phase inoculum of 5 × 10^5^ cfu/ml. Single colonies were suspended in MHB and were incubated for 2 h in a shaking water-bath; this was adjusted to the test inoculum using 0.5 of the McFarland climax. *Escherichia coli* reference strain ATCC 25922 was run in parallel in all experiments. Water-soluble amorphous powders of amikacin and tigecycline from Sigma-Aldrich (St. Louis, MO, USA); of colistin sulfate salt from AppliChem GmbH (Darmstadt, Germany); of meropenem from AstraZeneca (Cambridge, UK); and of rifaximin powder from Alfa Wassermann SpA (Bologna, Italy) were used. Owing to the poor solubility of rifaximin in water, the agent was first diluted in ethanol. To limit the excess use of ethanol, rifaximin was further diluted with 4% bile acid (Difco Oxgall, dehy-drated fresh bile; Becton Dickinson, Le Pont-de-Claix, France) and then added to the growth medium (MHB), as described previously^15^. Interpretation of results was done using the European Committee on Antimicrobial Susceptibility Testing (EUCAST) susceptibility breakpoints^37^.

### Time-kill assay

The killing effect of rifaximin over time was studied against 20 CRKP isolates. A log-phase culture (5 × 10^6^ cfu/ml) of each isolate was exposed over time in tubes with MHB at a final volume of 10 ml with or without rifaximin at concentrations of 500 μg/ml, 250 μg/ml and 100 μg/ml for 24 h. Rifaximin was dissolved as described above. At baseline, 2, 4, 6 and 24 h of incubation at 37 °C in a shaking water-bath a 0.1 ml aliquot from each tube was plated, after six serial 1:10 dilutions, onto MacConkey agar (Becton Dickinson). Bacterial counts were measured after 24 h incubation at 35 °C. The results were expressed as log_10_ cfu/ml. Any decrease in bacterial growth ≥ 3 log10 compared with the starting inoculum was considered as a bactericidal effect.

### Animal study

Animal experiments were conducted in the unit of animals for medical and scientific purposes of ATTIKON University General Hospital (Athens, Greece) according to EU Directive 2010/63/EU and to the Greek law 2015/2001, which incorporates the Convention for the Protection of Vertebrate Animals used for Experimental and Other Scientific Purposes of the Council of Europe (code of the facility EL 25BIO014, approval no. 1853/2015). All experiments were licensed from the Greek veterinary directorate under the protocol number 2027/09-04-2019. The study is reported in accordance with ARRIVE guidelines. We used 90 male and female C57Bl6 mice 7–8 weeks old. Mice were allowed to acclimate for seven days before starting the experiments. Mice were housed in individually ventilated cages, up to 5 mice per cage on a 12-h dark/light cycle and allowed free access to standard dry rodent diet and water. Analgesia was achieved with paracetamol suppositories.

We used a clinical blood isolate of a *Klebsiella pneumoniae* 87Β producing carbapenemase KPC-2 with MIC of amikacin, meropenem, colistin, tigecycline and rifaximin > 128, > 32, > 32, > 32 and 64 μg/ml respectively.

At the first stage we established a reproducible model of gut colonization by KPC. Mice were randomized in 3 groups (n = 10 in each group) receiving orally by a 12G gavage needle 200 μl of (a) 0.9% NaCl; (b) 40 mg/kg omeprazole^38^ diluted in 0.9% NaCl (Vianex, Athens, Greece) or (c) 50 mg/kg ampicillin^39^ (AppliChem) diluted in 0.9% NaCl once daily for 5 consecutive days. Then all mice were given orally once daily for 7 consecutive days 200 μl of 1 × 10^8^ cfu/ml KPC in 0.9% NaCl, so as to avoid intestinal irritation by MHB. On day 0, 3, 7 and 11 after the beginning of KPC-KP administration, stool samples of each mouse were collected into sterile tubes with 1 ml NaCl 0.9%.

Based on preliminary results showing that pre-treatment with ampicillin was necessary for gut colonization, further treatment experiments were done. In these experiments, 60 mice pre-treated with ampicillin for 5 days, were administrated orally using a gavage needle once daily for 7 consecutive days 200 μl of 1 × 10^8^ cfu/ml KPC-KP in 0.9% NaCl. Then, mice were treated with either 250 μl of 60 mg/kg rifaximin or vehicle three times per day for seven consecutive days^39^. Rifaximin was dissolved in 50 μl of ethanol and further diluted in 200 μl 4% bile. Vehicle was 50 μl of ethanol diluted in 200 μl 4% bile in MHB. On days 0, 3, and 7 after start of treatment, stool samples were collected into sterile tubes with 1 ml NaCl 0.9%. Ten mice from each group were sacrificed 3, 7 and 11 days after start of treatment. Sacrifice was done by the subcutaneous injection of 300 mg/kg ketamine. Under sterile conditions a midline abdominal incision was performed and segments of the duodenum (1 cm after the pyloric sphincter), of the ileum (1 cm before the ileocecal valve) and of the sigmoid (2 cm before the end of the rectum) were excised. They were washed with NaCl 0.9% to remove remaining stool and collected into sterile tubes with 1 ml NaCl 0.9%. In parallel, segments of the liver, of the spleen and of the right kidney were excised and collected into sterile tubes with 1 ml NaCl 0.9%. The samples were weighted and homogenized. One aliquot of 0.1 ml of the stool and tissue homogenates was diluted 1:10 into Mueller–Hinton broth six consecutive times; 0.1 ml of each dilution was plated onto CHROMID^®^ CARBA SMART agar (Biomerieux, Marcy-l'Étoile, France). After incubation for 24 h at 37 °C, the number of viable colonies was counted, and results were expressed as log_10_ of colony forming units per gram tissue (cfu/g).

### Statistical analysis

Quantitative variables were presented as mean ± standard error mean (SEM). Comparisons between groups were done using the Mann–Whitney U test. Any p value below 0.05 was considered statistically significant.
